# Biological Timing and Neurodevelopmental Disorders: A Role for Circadian Dysfunction in Autism Spectrum Disorders

**DOI:** 10.3389/fnins.2021.642745

**Published:** 2021-03-12

**Authors:** Ethan Lorsung, Ramanujam Karthikeyan, Ruifeng Cao

**Affiliations:** ^1^Department of Biomedical Sciences, University of Minnesota Medical School, Duluth, MN, United States; ^2^Department of Neuroscience, University of Minnesota Medical School, Minneapolis, MN, United States

**Keywords:** circadian rhythms, autism spectrum disorders, melatonin, cortisol, serotonin, clock genes, mTOR, sleep

## Abstract

Autism spectrum disorders (ASDs) are a spectrum of neurodevelopmental disorders characterized by impaired social interaction and communication, as well as stereotyped and repetitive behaviors. ASDs affect nearly 2% of the United States child population and the worldwide prevalence has dramatically increased in recent years. The etiology is not clear but ASD is thought to be caused by a combination of intrinsic and extrinsic factors. Circadian rhythms are the ∼24 h rhythms driven by the endogenous biological clock, and they are found in a variety of physiological processes. Growing evidence from basic and clinical studies suggest that the dysfunction of the circadian timing system may be associated with ASD and its pathogenesis. Here we review the findings that link circadian dysfunctions to ASD in both experimental and clinical studies. We first introduce the organization of the circadian system and ASD. Next, we review physiological indicators of circadian rhythms that are found disrupted in ASD individuals, including sleep–wake cycles, melatonin, cortisol, and serotonin. Finally, we review evidence in epidemiology, human genetics, and biochemistry that indicates underlying associations between circadian regulation and the pathogenesis of ASD. In conclusion, we propose that understanding the functional importance of the circadian clock in normal and aberrant neurodevelopmental processes may provide a novel perspective to tackle ASD, and clinical treatments for ASD individuals should comprise an integrative approach considering the dynamics of daily rhythms in physical, mental, and social processes.

## Introduction

Circadian rhythms are evolved as a result of the axial rotation of the earth and have been observed in almost all living organisms including human beings. The approximately 24 h rhythms are intrinsically driven by circadian clocks but are entrained by environmental cues such as light (Reppert and Weaver, 2002). Many neurophysiological processes exhibit robust daily fluctuations in their functional states. In humans, language, learning, memory, and social behavior adapt to the sleep–wake cycles, and the performance in all these activities exhibits daily fluctuations (Amir and Stewart, 2009). The functional significance of the circadian clock is being increasingly appreciated as circadian dysfunctions have been linked to an increasing number of human diseases including metabolic syndromes, cardiovascular diseases, diabetes, and cancer (Rijo-Ferreira and Takahashi, 2019). Anomalies in timing have been observed in neurological and psychiatric diseases including seasonal affective disorders, bipolar disorder, and schizophrenia, etc. (Wehr et al., 2001; Wulff et al., 2012; Logan and McClung, 2019). In neurodegenerative diseases such as Alzheimer’s disease, the disruption of daily activity rhythms is often associated with or even precedes underlying pathophysiological changes in the brain (Duncan, 2020). In fact, disruption of daily rhythms is the leading cause of institutionalization of individuals with Alzheimer’s disease (Musiek et al., 2015). Thus, a key role for circadian regulation/deregulation in neurological and psychiatric disorders is emerging in recent decades.

Autism spectrum disorders (ASDs) are a compilation of neurodevelopmental disorders defined by behavioral abnormalities (Mughal et al., 2020; American Psychiatric Association DSM-5). Growing evidence indicates dysfunction of the endogenous circadian system is associated with the neural dysfunctions prevalent in the development of ASD. Studies on the circadian clock and sleep in ASD improve our understanding of its pathogenesis and inspire potential chronotherapeutic strategies to treat or prevent the diseases. In this review, we discuss the involvement of the circadian timekeeping system in the development and functionality of the nervous system, and summarize evidence indicating underlying links between the circadian clock and ASD. We first introduce the organization of the circadian system and ASD. Next, we review physiological parameters of endogenous rhythms that are found disrupted in ASD patients, including the sleep/wake cycle, and the daily oscillations of the circadian biomarkers melatonin, cortisol and serotonin. Finally, we review evidence indicating underlying links between circadian dysfunction and ASD pathogenesis, including epidemiology, human genetics, and the mTOR pathway.

### The Circadian Timekeeping System

The term “circadian” was originally coined by Franz Halberg from the Latin root *circa* meaning “around” and *diem* meaning “day.” Circadian rhythms refer to the approximately 24 h rhythms that are found in a variety of physical, mental, or behavioral processes (Halberg, 1969; Pittendrigh, 1993). The rhythms are endogenously driven by circadian clocks, which are oscillating proteins in cells that are found in nearly all living organisms (Rosbash, 2009). A variety of physiological events are regulated by circadian clocks and exhibit circadian rhythms, including the sleep–wake cycles, core body temperature, blood pressure, hormone secretion, and cognition (Patke et al., 2020). Circadian rhythms are found at every level of the organization of life: cellular, tissue, organ, and organismal level (Takahashi et al., 2008).

**FIGURE 1:**
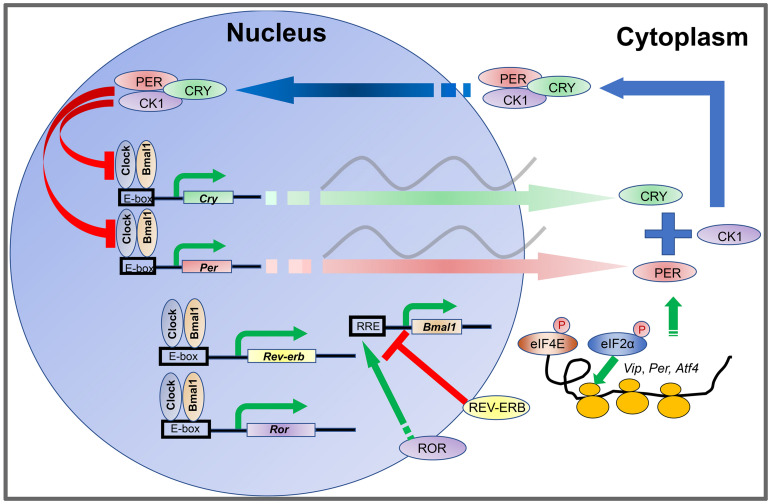
Transcription-translation feedback loops (TTFLs) in the mammalian circadian clock. The CLOCK and BMAL1 proteins are activators and form a heterodimer to bind to E-box enhancers in the promoters of *Per* and *Cry* genes. PER and CRY proteins are synthesized during the day and form a protein complex which accumulates in the cytoplasm during the afternoon and evening. Upon reaching certain level, the PER-CRY complexes translocate into the cell nucleus during the nighttime and block the activities of the CLOCK: BMAL1 heterodimer to inhibit their own gene transcription. In addition, the CLOCK: BMAL1 complex also promotes the transcription of *Rev-erb* and *Ror*. REV-ERB in turn inhibits *Bmal1* transcription whereas ROR promotes *Bmal1* transcription. The abundance of PER proteins is controlled at the level of mRNA translation by rhythmic phosphorylation of eIF4E. Phosphorylation of eIF2α promotes translation of *Atf4*. ATF4 directly activates *Per2* transcription. At the posttranslational level, levels of PER and CRY protein are regulated by phosphorylation and ubiquitination-mediated protein degradation CKI phosphorylates PER. Phosphorylation of PER and CRY proteins promotes their degradation and speeds up the clock.

The oscillations of the cellular clock are driven by transcriptional-translational feedback loops (TTFLs) (Rosbash, 2009). In mammals, TTFLs are driven by rhythmic oscillations of about a dozen clock genes and their protein products (Figure 1), including two *Period* genes (*Per1* and *Per2*), two *Cryptochrome* genes (*Cry1* and *Cry2*), *Clock, Bmal1, Rev-erbα/β*, *Rorα/β/γ*, and *CkIε/δ* (Takahashi et al., 2008). The CLOCK and BMAL1 proteins are activators and form a heterodimer to bind E-box enhancers in the promoters of *Per* and *Cry* genes. PER and CRY proteins are synthesized during the day and form a protein complex which accumulates in the cytoplasm during the afternoon and evening. Upon reaching a certain level, the PER-CRY complexes translocate into the cell nucleus during the nighttime and block the activities of the CLOCK: BMAL1 heterodimer to inhibit their own gene transcription (Shearman et al., 2000; Ramanathan et al., 2006; Yan et al., 2020). In addition, the CLOCK: BMAL1 complex also promotes the transcription of *Rev-erbα/β* and *Rorα/β/γ*. REV-ERBα/β in turn inhibits *Bmal1* transcription whereas ROR*α/β/γ* promotes *Bmal1* transcription (Preitner et al., 2002). In this way, the CLOCK: BMAL1 heterodimer is a self-regulator.

The abundance of PER proteins is also controlled at the level of mRNA translation by an eIF4E-dependent mechanism. Rhythmic phosphorylation of eIF4E by the mitogen-activated protein kinase-interacting kinases (MNKs) promotes mRNA translation of *Per1* and *Per2* (Cao et al., 2015). At the posttranslational level, levels of PER and CRY proteins are regulated by phosphorylation and ubiquitination-mediated protein degradation (Hirano et al., 2013; Yoo et al., 2013). CKIε and CKIδ phosphorylate PER (Lee et al., 2001; Meng et al., 2008; Etchegaray et al., 2009; Lee et al., 2011), whereas AMPK phosphorylates CRY (Lamia et al., 2009). Phosphorylation of PER and CRY proteins promotes their degradation and speeds up the clock. Although intracellular clock mechanisms are thought to be conserved in different cells, intercellular coupling mechanisms are unique between neurons and glial cells in the suprachiasmatic nucleus (SCN) and confer robustness and precision to the SCN clock (Aton and Herzog, 2005; Hastings et al., 2018). When SCN cells are isolated, the cell autonomous oscillations are poorly organized (Welsh et al., 1995; Herzog et al., 1998; Patton et al., 2016). Numerous body clocks are orchestrated by the SCN pacemaker in the hypothalamus, which is a pair of tear-drop-like structures in the inferior portion of the brain composed of ∼20,000 neurons (Moore et al., 2002; Hastings et al., 2018). The neurons express the neuropeptide vasoactive intestinal polypeptide (VIP) and gastrin-releasing peptide (GRP) in the core (ventral) region of the SCN, and arginine vasopressin (AVP) in the shell (dorsal) region. The astrocytes are regulatory cells to the neurons, and primarily utilize the neuro-excitatory molecule glutamate at night to inhibit SCN neuron activity (Brancaccio et al., 2017).

**FIGURE 2:**
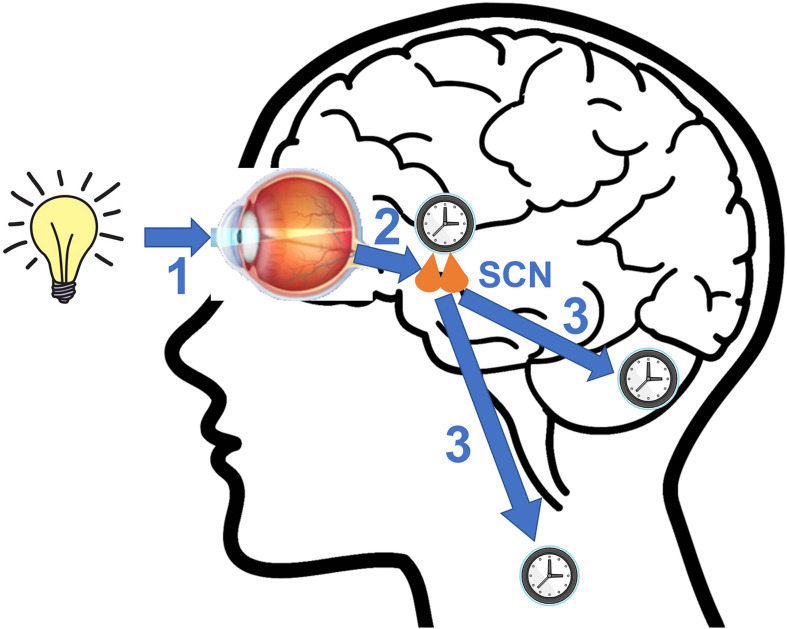
A diagram illustrating key steps involved in photic entrainment of the circadian system. **(1)** Ambient light stimulates intrinsically photosensitive retinal ganglion cells (ipRGCs) in the retina. **(2)** The axons of ipRGCs travel via the retinohypothalamic tract (RHT) to form synaptic connections with the core neurons of hypothalamic suprachiasmatic nucleus (SCN). Glutamate and pituitary adenylate cyclase activating polypeptide (PACAP), among other neurotransmitters are released at the synapses of the RHT terminals to the SCN neurons. Synaptic activities induce clock gene expression and reset the SCN clock. **(3)** SCN sends rhythmic outputs to other brain regions and peripheral oscillators to reset their rhythms.

The circadian clocks are entrained by external signals called zeitgebers to synchronize themselves with the ever-changing environment (Figure 2). The SCN utilizes light as its primary zeitgeber. SCN receives photic information from the intrinsically photosensitive retinal ganglion cells (ipRGCs) in the retina (Berson et al., 2002). The ipRGCs express the photopigment melanopsin and their axons form the retinohypothalamic tract (RHT) that terminates in the SCN. The RHT pathway is separated from the image forming visual pathway (Provencio et al., 2002; Peirson and Foster, 2006). The RHT terminals form direct synaptic connections with the core SCN neurons that express the neuropeptides VIP or GRP. Upon photic stimulation at night, RHT terminals release glutamate and the neuropeptide pituitary adenylate cyclase activating polypeptide (PACAP) that are the neurotransmitters functioning to evoke clock gene expression and reset the SCN clock by regulating intracellular signaling pathways (Obrietan et al., 1998; Butcher et al., 2002; Hannibal, 2002).

Besides light, non-photic inputs (electrical stimulation, odor, etc.) can also influence the SCN through two brain regions, the intergeniculate leaflets (IGL) and the dorsal/median raphe nucleus (DRN/MRN) (Rusak et al., 1989; Meyer-Bernstein et al., 1997). The afferent pathway from the IGL is the geniculohypothalamic tract (GHT), and the DRN/MRN communicates with the SCN through serotonergic neurons (Meyer-Bernstein and Morin, 1996; Moga and Moore, 1997). Besides these inputs, other external cues such as social activities, exercise, and temperature have been examined as zeitgebers to the sleep-wake cycle in adult humans, but there are critiques of the role for social zeitgebers beyond their role of light regulation (Korczak et al., 2008). The SCN communicates internally with peripheral tissues through neural and endocrine outputs, i.e., electrical signals, neurotransmitters, and hormones (Kalsbeek et al., 2006). The SCN resets the peripheral clocks via these output signals. The peripheral clocks can also be reset by extrinsic and intrinsic cues that are relevant to their physiological functions. For example, adrenal hormones and feeding schedule are of notable importance to liver clock gene rhythms (Su et al., 2016). The timing of physical activities is a cue for the skeletal muscle clock (Wolff and Esser, 2012). The peripheral clocks regulate local physiology and help to orchestrate the organismal function by synchronizing rhythms in various systems. Thus, by synchronizing with the environmental light-dark cycles, the SCN clock orchestrates rhythms in different systems and coordinates various physiological processes and systemic well-being.

### Autism Spectrum Disorders (ASDs)

Autism spectrum disorders are a group of developmental disabilities diagnosed by core behavioral symptoms including trouble with social interaction, abnormal communication skills, and atypically restricted, stereotyped, repetitive behaviors (American Psychiatric Association DSM-5, 2013). Children are now commonly diagnosed by 3 years of age, which is earlier than in the past (Mazurek et al., 2014). Clinical symptoms exhibited by autistic children are not uniform. Children with autism have social developmental problems and exhibit interest toward repetitive behavioral processes (Bodfish et al., 2000). Developmental deficits in ASDs have been confirmed by studies finding abnormalities in both prenatal and postnatal brain development (Carper et al., 2002; Hazlett et al., 2005; Bonnet-Brilhault et al., 2018). Their delay in development of communication and restricted interests is thought to be correlated to the severity of the anomalies in the brain (Anderson et al., 2009). ASD is often accompanied by intellectual disability and hyperactivity. In addition, children with ASD commonly exhibit comorbid medical conditions, including abnormal tactile sensation, food selectivity, and sleep disruption (Baranek et al., 2006; Ben-Sasson et al., 2007; Souders et al., 2009). Currently there is no unified theory to explain all core and comorbid abnormalities in ASD children.

The incidence of ASD has dramatically increased around the globe in the past 50 years (World Health Organization, 2017). For example, according to a British study, autism incidence rate was 4.5 per 10,000 children in the 1960s (Lotter, 1966). In the 1980s, the prevalence of autism was between 5 and 12 in 10,000 persons (Gillberg et al., 1991). In the U.S., the frequency of the autism has risen from 3 per 10,000 individuals in 1991–1992 to 53 per 10,000 children in 2003–2004 (Gurney et al., 2006). According to estimates from CDC’s Autism and Developmental Disabilities Monitoring Network, about 1 in 54 children are diagnosed with ASDs in 2014^1^. Notably, the diagnostic criteria for ASD was changed in the DSM-5 published in 2013, and an expanded group of disorders are now classified as ASDs (American Psychiatric Association DSM-5, 2013). ASDs now include several conditions that used to be diagnosed separately including autistic disorder, pervasive developmental disorder not otherwise specified (PDD-NOS), and Asperger syndrome. The high prevalence of ASDs is accompanied by unprecedented social and economic burdens on the affected families and society (Newschaffer et al., 2007). The yearly total costs for children with ASD were estimated to be between $11.5 and $60.9 billion in the U.S. The clinical expenses of autism children are comparatively higher than normal children and are ten times greater than the costs of normal children’s medical expenditure (Mandell et al., 2006). Children with ASD cost more than those without ASD by $4,110–$6,200 per year. Thus, there is an urgent need to find novel therapeutic strategies to tackle these diseases.

**FIGURE 3:**
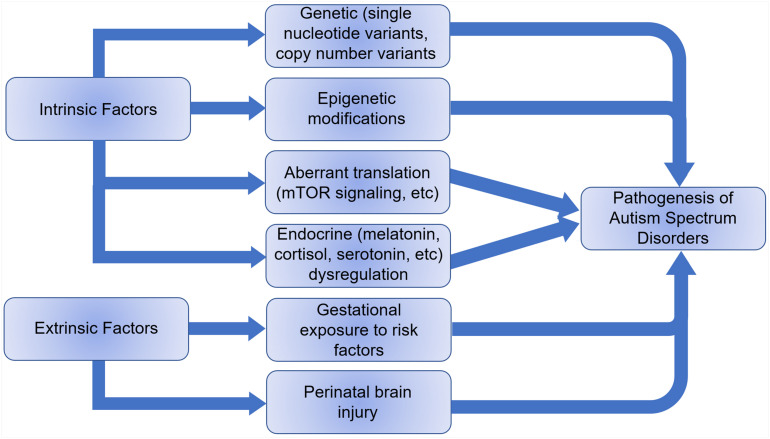
Intrinsic and extrinsic factors that can lead to the pathogenesis of autism spectrum disorders.

The etiology of ASD remains elusive, but it is thought to be a combination of extrinsic and intrinsic factors (Figure 3). Less than 20% of ASDs have an identifiable genetic origin, whereas over 75% of cases are idiopathic, suggesting a multifactorial etiology (Abrahams and Geschwind, 2008). The discovery of single nucleotide variants (SNVs) and copy number variants (CNVs) in genes associated with ASD supports the claim for a genetic basis of ASD etiology and over 1200 risk genes have been identified (Xiong et al., 2019^2^). In addition, epigenetic and immunological factors are also speculated as possible causes of autism (Lee et al., 2015; Sun et al., 2016). An increased risk of ASD with advanced paternal age coupled to an increased rate of DNA methylation abnormalities in older fathers at multiple imprinted gene loci suggests an epigenetic association (Kong et al., 2012; Smith et al., 2013). Animal models have shown transgenerational aberrant DNA methylation and histone modifications with abnormal neurodevelopment as a result of abnormal nutrition, stress and drugs, as well as transplacental psychiatric medication affecting GABAergic, dopaminergic, serotonergic, and glutamatergic pathways (Franklin et al., 2010; Morgan and Bale, 2011). Besides genetic and epigenetic factors, development of ASD can be influenced by embryonic exposure to detrimental environmental factors including pollution, maternal stressors, etc. (Becerra et al., 2013; Volk et al., 2013; Walder et al., 2014). Perinatal brain injury, especially cerebellar injury, can also contribute to autism development (Singh et al., 2016).

Multiple theories have been proposed regarding the neural mechanisms underlying ASD pathogenesis, but no theory has convincingly integrated the diverse behavioral dysfunctions in autism. Aberrant neurotransmission of dopamine, glutamate, serotonin, oxytocin/vasopressin and GABA have all been implicated in the development of ASD (Modahl et al., 1998; Blatt et al., 2001; Chandana et al., 2005). A number of studies suggest that glutamate systems are dysfunctional in ASD (Purcell et al., 2001; Shinohe et al., 2006; Rojas, 2014). The dysfunction of the GABAergic system, synthesized from glutamate, has been suggested to result in impaired cognitive and motor function as well as seizure disorder, a comorbidity of autism (Russo, 2013; Rojas, 2014). Excessive dopamine receptor DRD1a activation has been shown to elicit autistic behaviors in mouse models (Lee et al., 2018). The increased prevalence of ASD in recent decades cannot be simply explained by reclassification and increased diagnosis. Neither can it be completely ascribed to genetic factors that cause the disease. Apparently, a combination of extrinsic and intrinsic factors should be investigated in the development of autism.

## Disruption of Sleep and Daily Rhythms in ASD

### Sleep Problems in ASD

Sleep is a conserved physiological process in animals that is critical for brain development and maturation. Suboptimal sleep can have adverse effects on children’s cognitive functions including attention, memory, mood regulation, and behavior (Pilcher and Huffcutt, 1996; Belenky et al., 2003). Children with ASD exhibit sleep problems at a higher rate than children with other developmental disorders as well as typically developing children (Owens et al., 2000; Cotton and Richdale, 2006; Johnson and Zarrinnegar, 2021). Repeated sleep disruption adversely affects the process of neural development in ASD children, whereas impaired neurodevelopment further exacerbates the sleep problem in ASD.

It is estimated that 50∼80% ASD children have sleep problems, compared to less than 30% in the general children population (Souders et al., 2017). Prolonged sleep latency, frequent waking at night, alterations in sleep architecture, unusual morning arousal and reduction in total sleep duration are commonly found in children with autism (Richdale, 1999; Polimeni et al., 2005). A reduced percentage of REM sleep and higher percentage of slow-wave sleep has been observed in association with ASD (Buckley et al., 2010). There is also a report of a higher rate of REM sleep behavior disorder in autistic children (Thirumalai et al., 2002). Atypical REM sleep patterns indicate disruption in central nervous system maturation and neuronal network organization, as well as atypical synapse homeostasis involved in sleep–wake function (Buckley et al., 2010). Notably, sleep problems may differ between different types of ASD patients. In a study by Richdale and Prior (1995), it was found that low functioning (IQ < 55) ASD individuals showed increased naps, earlier time going to sleep, increased sleep latency, increased sleeping time at night, and increased total sleeping time over 24 h compared to controls. By contrast, high functioning (IQ > 55) ASD individuals exhibited increased sleep latency, decreased total night sleep, increased length of waking episodes at night, and earlier wake time. A more detailed review on sleep problems in ASD has been published recently (Karthikeyan et al., 2020).

The possible causes for the sleep problems in ASD can be classified into four categories. (1) Synaptic protein abnormalities. Sleep and synaptic functions are tightly interconnected. Sleep relies on normal functionality of complex neural circuitries with synapses as the connectors between neurons (Scammell et al., 2017). Proper sleep is essential for synaptogenesis and synaptic plasticity (Cirelli and Tononi, 2019). The disruption of the Neurexin/Neuroligin/Shank synaptic protein complex has been found to be involved in ASD (Jamain et al., 2003; Durand et al., 2007). Neuroligins and Neurexins are synaptic proteins of excitatory glutamatergic and inhibitory GABAergic synapses. Neuroligin-1/3/4 are confined to glutamatergic synapses whereas neuroligin-2 is specific to GABAergic synapses (Graf et al., 2004; Varoqueaux et al., 2004). Neurexins, encoded by *Nrxn 1/2/3*, are a family of presynaptic cell adhesion proteins that interact with Neuroligins to connect neurons at the synapse. Mutation of Shank3, a gene encoding a scaffolding protein on the postsynaptic membrane that tethers Neuroligins and regulates dendritic organization, has been shown to be associated with autism (Durand et al., 2007). Furthermore, a *de novo* deletion on chromosome 2p16 encoding Neurexin-1 was identified in ASD (Szatmari et al., 2007). Interestingly, Neuroligins, Neurexin, and Shank3 have all been shown to regulate the sleep architecture and clock gene expression in mouse models (El Helou et al., 2013; Tong et al., 2016; Seok et al., 2018; Ingiosi et al., 2019). It is possible that dysregulated synaptic proteins link sleep disorders to the development of autism. (2) Sensory dysregulation and increased arousal (Wiggs and Stores, 2004; Souders et al., 2009). This can be explained by two hypotheses, cognitive arousal and physiological arousal. Cognitive arousal, which is caused by increased cognitive activities due to increased anxiety in ASD, can increase the sleep latency. Physiological arousal is caused by increased responses to environmental stimuli due to low sensory thresholds in children with ASD, and can result in difficulty falling or staying asleep (Mazurek and Petroski, 2015). (3) Abnormal sleep-regulating hormones. Abnormal levels of hormones such as melatonin in ASD will be discussed in “Disruption of Circadian Biomarkers in ASD.” (4) Circadian sleep disruptions. Mutations in genes that regulate circadian timing can also cause changes in the timing and duration of sleep in ASD and will be discussed in “Clock Gene Polymorphisms in ASD.” It is important to recognize the possible overlaps in causes, and their additive or amplified effects on the complex sleep problems in ASD.

### Disruption of Circadian Biomarkers in ASD

In clinical studies, levels of traditional circadian biomarkers including melatonin and cortisol are measured from biological specimens such as blood, urine, and saliva at different times of day in order to assess the functions of the body clock. Melatonin is a pineal hormone with daily rhythmic synthesis that peaks at night and is suppressed by light during the day (Socaciu et al., 2020). Cortisol is a sterol hormone that peaks in the early morning and falls throughout the day (Russell and Lightman, 2019). Serotonin is a monoamine neurotransmitter and also the intermediate product to synthesize melatonin (Comai et al., 2020). Here we discuss how levels and daily rhythms of these biomarkers are changed in ASD, and how abnormalities in these hormones may in turn contribute to neural dysfunctions in ASD individuals. The abnormalities of these biomarkers have been summarized in Table 1.

**TABLE 1:** Circadian dysfunctions in ASD.

ASD patients and control	Circadian biomarkers	Findings	References
**Age:** Mean = 9 years **Number(sex):** 19 (M), 3 (F) **Control:** Six adults (mean age = 30 years), 5 (M) and 1 (F); 27 children (mean age = 9 years), 15 (M) and 12 (F) **Other factors:** 15 highly developed and 7 poorly developed ASD cases based on IQ 60	Cortisol in saliva and blood	Abnormal diurnal rhythm of salivary cortisol (higher peak in the morning) and lower response in dexamethasone suppression test in ASD vs. control, especially in poorly developed cases	Hoshino et al., 1987
**Age:** 4–19 years, mean = 10.2 years **Number(sex):** 30 in total, no sex data **Control:** 106 children, aged 1–19 years, mean = 9.7 years; 17 adults, aged 20–55 years, mean = 35.5 years	Serotonin in blood	(1) Summer serotonin levels in ASD significantly are lower compared to other seasons. (2) Average serotonin level in ASD is significantly higher than controls.	Badcock et al., 1987
**Age:** 4–14 years, mean = 8.3 years, **Number(sex):** 14 (M), 4 (F) **Control:** 16 (M), 3 (F)	Cortisol in urine	Increased cortisol levels at all times of day, particularly morning to mid-afternoon	Richdale and Prior, 1992
**Age:** Mean = 18 years **Number(sex):** 10 in total, no sex data **Control:** 15 parents, 1 grandparent, 9 siblings, and 10 unrelated healthy individuals **Other factors:** Control were significantly older than autism group	Melatonin in urine	Increased daytime melatonin level and ratio of daytime/nighttime melatonin levels compared to controls	Ritvo et al., 1993
**Age:** 16–30 years **Number(sex):** 10 (M) **Control:** 5 matched in age and weight	Melatonin in blood	(1) Melatonin levels in ASD higher during the day and lower at night vs. controls (2) No differences in cortisol levels	Nir et al., 1995
**Age:** 3–23 years, mean = 9.2 years **Number(sex):** 42 (M), 20 (F) **Control:** 91 in total, aged 2–16 years, age and sex matched **Other factors:** Relatives of autism patients were also examined for serotonin levels	Serotonin in blood	(1) Higher serotonin levels in ASD vs. control above age 16 (2) No difference in serotonin levels between ASD and control below age 16 (3) Distribution of serotonin levels significantly more variable in ASD than control (4) Serotonin levels in control decrease with age, while serotonin levels in ASD is independent of age	Leboyer et al., 1999
**Age:** Mean = 8.5 years **Number(sex):** 12(M) **Control:** 10 (M), mean age = 9.2 years **Other factors:** Groups were matched on age and gender but not on IQ. Mean IQ of autism group = 77, and mean IQ of normal group = 114	Cortisol in saliva	(1) No significant difference in mean cortisol daily variation between children with autism and typically developing children (2) Children with autism showed significantly increased response to a non-social stressor (mock MRI), while typically developing children showed no response in cortisol level	Corbett et al., 2006
**Age:** 14.8 ± 7 years **Number(sex):** 29 (M), 14 (F) **Control:** 45 (M), 30 (F), sex and age matched. Thirty four parents of ASD patients were also examined.	Asmt mutations, melatonin and serotonin in blood and platelets	(1) Non-conservative variations of Asmt (the gene encoding the last enzyme of melatonin synthesis) identified in ASD families but not in controls. Two polymorphisms located in the promoter were more frequent in ASD compared to controls associated with a decrease in ASMT transcripts in blood cell lines (2) Decreased in ASMT activity and melatonin levels in individuals with ASD and damped melatonin daily rhythms in ASD (3) Increased serotonin levels in ASD and their parents compared to controls (4) Poor sleep efficiency and higher arousal index but normal REM and slow wave sleep in patients with ASMT mutations	Melke et al., 2008
**Age:** Mean = 9.08 years, Range = 6.5–12 years **Number(sex):** 21(M), 1(F) **Control:** 19(M), 3(F) **Other factors:** Cortisol levels were measured in anticipation and response to a stressful event (mock-MRI)	Cortisol in saliva	(1) Children with autism showed consistently higher cortisol levels in the evening (2) Diurnal variations of cortisol are more inconsistent in autism individuals	Corbett et al., 2008
**Age:** Mean = 9.1 years **Number(sex):** 13(M), 2(F) **Control:** 21(M), 4(F), aged 6–12 years	Cortisol in saliva	No significant difference in the cortisol awakening response between individuals with high functioning autism and controls	Zinke et al., 2010
**Age:** 2–5 years, mean = 3.75 years **Number(sex):** 22(M), 4(F) **Control:** 23(M), 3(F), mean age = 3.3 years	Cortisol in saliva	(1) Moderately increased mean cortisol secretion levels in autism children upon waking compared to controls (not statistically significant *p* > 0.05) (2) Mildly increased mean cortisol in autism children during daytime and evening compared to controls (not statistically significant *p* > 0.05)	Kidd et al., 2012
**Age:** Mean = 10.3 years **Number(sex):** 47 in total, 35 autistic disorder, 10 Asperger syndrome, five pervasive development, no sex data included **Control:** 50 in total, mean = 9.9 years	Cortisol in saliva	No differences in cortisol levels at any given time point for ASD children when compared with controls	Corbett and Schupp, 2014
**Age:** Mean = 10.2 years **Number(sex):** 30(M), 6(F) **Control:** 23(M), 4(F), mean = 9.71 years	Cortisol in saliva	(1) Higher overall cortisol levels in ASD than control (2) Higher cortisol levels in ASD in the evening compared to controls (3) Flatter diurnal cortisol rhythm in some ASD children	Tomarken et al., 2015
**Age:** LFASD mean = 9.23 years, HFASD mean = 9.38 years **Number(sex):** LFASD 13(M), HFASD 16(M) **Control:** 14(M), mean age = 9.36 years	Cortisol in saliva	(1) Children with low functioning ASD (LFASD) demonstrated higher cortisol levels at morning, afternoon, and evening compared with children with high functioning ASD (HFASD) and normal children (2) Lower cortisol levels in HFASD individuals in the morning than typically developing individuals	Putnam et al., 2015
**Age:** Mean = 7.51 years **Number(sex):** 35(M), 8(F) **Control:** 30(M), 10(F), mean = 7.83 years	Cortisol in saliva and serotonin in blood	(1) Elevated cortisol levels in ASD compared with control (2) Elevated serotonin levels in ASD compared with control (3) Flattened cortisol diurnal rhythms in ASD compared with control	Yang et al., 2015

#### Melatonin

Melatonin is a neurohormone synthesized in the pineal gland. Melatonin levels are normally higher at night than during the day in both nocturnal and diurnal animals. Melatonin induces sleep and resets the SCN circadian clock (McArthur et al., 1991; Zhao et al., 2019). Sleep phase and duration is determined by the phase of the melatonin cycle suggesting a key role for melatonin in regulating the sleep–wake cycle (Lockley et al., 1997). Melatonin has direct effects on the SCN circadian clock through its two G protein coupled receptors MT1 and MT2. MT1 is the high affinity receptor responsible for acute suppression of neuronal firing and MT2 is the low affinity receptor required for efficient phase-shifts (Liu et al., 1997; Jin et al., 2003). Binding of melatonin to MT1 and MT2 indirectly regulates clock gene expression by inhibition of adenylate cyclase, and inhibition of PKA due to reduction of cAMP. The G protein coupled receptors also directly inhibit phosphorylation of cAMP response element-binding protein (CREB) (Ross et al., 1996; von Gall et al., 2002). CREB inhibition causes decreased expression of clock proteins PER1 and PER2 and attenuates photic entrainment of the circadian clock (Lee et al., 2010). Melatonin signaling disruption has been linked to sleep disorders such as insomnia, and has been reported in neurological and psychiatric conditions such as Parkinson’s disease and depression (Claustrat et al., 1984; Adi et al., 2010). There is also evidence that melatonin is involved in neural differentiation, and its dysfunction in ASD individuals could contribute to their non-typical development (Shu et al., 2016).

Melatonin is the most well documented circadian biomarker associated with ASD. Lower levels of melatonin and its major metabolite, urinary 6-sulfatoxymelatonin, have been found in the urine, serum, and plasma of ASD individuals (Nir et al., 1995; Tordjman et al., 2005; Melke et al., 2008). Low melatonin amplitude and a delayed melatonin rhythm have been associated with increased sleep problems in ASD children (Kulman et al., 2000; Melke et al., 2008). Excretory levels of the metabolite 6-sulphatoxymelatonin were decreased in a group of 50 autistic children and these decreased concentrations were associated with their verbal and play abilities (Tordjman et al., 2005). Seizure comorbidities and electroencephalogram (EEG) discrepancies in autism individuals have been associated with the aberrant phase cycles of melatonin (Nir et al., 1995). Increased levels of melatonin have been found during the daytime in small samples of autistic children, whereas no significant difference was reported in nighttime concentration (Ritvo et al., 1993; Kulman et al., 2000). Low overall levels of melatonin and a sharp increase in concentration during the daytime was reported in a study of 14 autistic children (Kulman et al., 2000). Interestingly, 10 of the 14 autistic individuals in the study exhibited no observable daytime rhythmic changes in their blood melatonin levels (Kulman et al., 2000). Unusual patterns of melatonin in both amplitude and phase suggests fundamental impairments of the body circadian clock. As the level of melatonin is in general decreased in ASD individuals, melatonin supplements at night before bedtime may help with the sleep problems in ASD. In one study, melatonin administered 30 min before bedtime improved sleep latency in ASD children (Malow et al., 2012).

The mechanisms underlying melatonin abnormalities in ASD remain elusive and is a topic still under investigation. It is unclear whether the total amount of melatonin is reduced in a circadian period or if the phase has been altered by the abnormal circadian clock (Tordjman et al., 2005). There may also be abnormalities in melatonin synthesis, regulation, or receptor binding and efficacy in ASD. There are three main G-protein coupled receptors (GPCR) receptors involved in melatonin signaling: *MNTR1A* (MT1), *MNTR1B* (MT2), and the orphan receptor *GPR50*, which has no affinity for melatonin, but inhibits melatonin signaling when bound to MT1 (Chaste et al., 2010). When individuals with ASD were screened for mutations in the genes encoding melatonin receptors, no significant difference was found compared to controls, indicating that abnormal melatonin production rather than abnormal receptor function may be involved in ASD. Thus, rectifying melatonin levels using exogenous melatonin is a plausible therapeutic strategy. Indeed, clinical evidence exists demonstrating high efficacy of melatonin treatment for ASD individuals with sleep disruption (Chaste et al., 2010; Malow et al., 2012).

**FIGURE 4:**
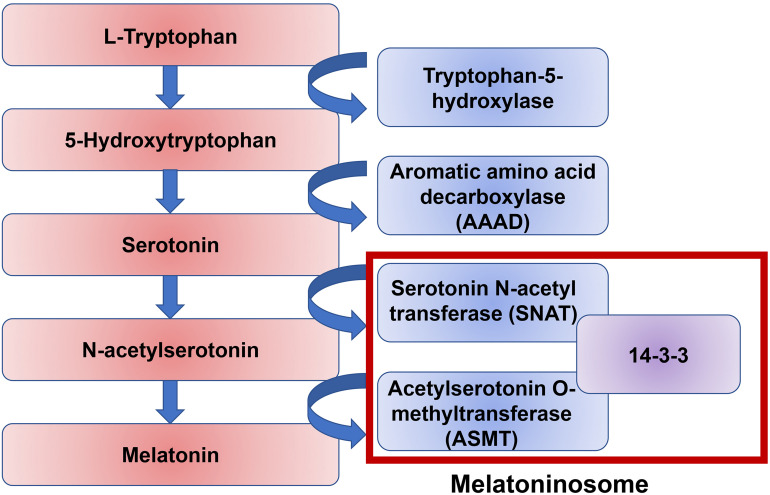
A pathway of melatonin biosynthesis. Melatonin synthesis is the result of the amino acid tryptophan through the intermediate neurotransmitter serotonin. The conversion of serotonin to melatonin is mediated by two enzymes, serotonin *N*-acetyl transferase (SNAT) and acetylserotonin *O*-methyltransferase (ASMT). The protein 14-3-3 mediates both of these stepwise interactions.

It is likely that melatonin disruption in a significant number of ASD individuals is due to dysfunction of melatonin synthesis. There are two enzymatic steps in the conversion of serotonin into melatonin: the conversion of serotonin to *N*-acetylserotonin by the enzyme Serotonin *N*-acetyl transferase (SNAT), and the conversion of *N*-acetylserotonin into melatonin by the enzyme Acetylserotonin *O*-methyltransferase (ASMT) (Figure 4). 14-3-3 is a family of conserved regulatory proteins that bind to a variety of signaling proteins. It has been proposed interaction of the protein 14-3-3 with SNAT, and more importantly 14-3-3 with ASMT, is necessary for melatonin synthesis (Obsil et al., 2001; Maronde et al., 2011). The protein miR-451, a known suppressor of 14-3-3, was elevated in ASD individuals (Pagan et al., 2017). An increasing body of evidence supports a disruption of the 14-3-3/ASMT/SNAT ‘melatoninosome’ in ASD individuals (Obsil et al., 2001; Maronde et al., 2011). Slow metabolization of melatonin may lead to accumulation of melatonin in the body and cause sleep problems. The liver cytochrome P450 enzyme, CYP1A2, has been demonstrated to be the primary metabolizing enzyme of melatonin in the liver (Facciolá et al., 2001). In the small number of ASD individuals where exogenous treatment with melatonin loses effectiveness, high level of melatonin was found around noon. It has been hypothesized this is due to a single nucleotide polymorphism (SNP) in CYP1A2 (Braam et al., 2013). While melatonin treatment has been shown to be effective in treating sleep–wake difficulties in ASD individuals, more research is necessary to elucidate the precise role of melatonin and its dysregulation in ASD.

#### Cortisol

**FIGURE 5:**
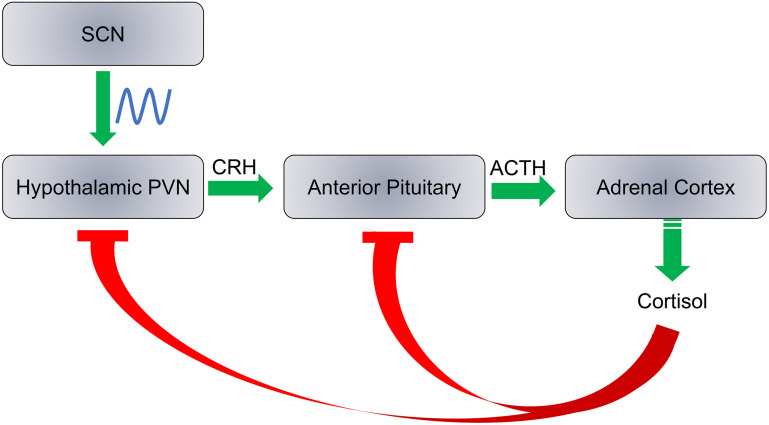
SCN regulates the hypothalamic-pituitary-adrenocortical axis. Cortisol production is regulated by three steps: (1) Release of corticotropin releasing hormone (CRH) by the hypothalamic paraventricular nucleus (PVN); (2) the adrenocorticotropic hormone (ACTH) is released from the anterior pituitary; and (3) Cortisol is synthesized and released from the adrenal cortex. Cortisol inhibits CRH and ACTH release at a feedback mechanism. The SCN has direct neural inputs into PVN and controls the daily rhythms of cortisol levels.

Cortisol production is a result of a cascade response along the hypothalamic-pituitary-adrenocortical (HPA) axis in three major steps with negative feedback at each step: (1) Corticotropin releasing hormone (CRH) is released by the paraventricular nucleus (PVN) of the hypothalamus; (2) Adrenocorticotropic hormone (ACTH) is released from the anterior pituitary; and (3) Cortisol is released from the adrenal cortex (Figure 5). Each hormone is released in response to the action of the preceding hormones action on its respective target tissue. The HPA axis regulates hormonal stress response. Daily cortisol levels exhibit robust oscillations as PVN is innervated by the SCN. Here we discuss circadian rhythm abnormalities of cortisol in ASD individuals and whether these abnormalities are associated with ASD as a causative factor that may exacerbate traits of the disorder, or simply a result of ASD symptoms.

The PVN receives direct neural input from the hippocampus, amygdala, prefrontal cortex, and SCN (Gray et al., 1989; Boudaba et al., 1996; Li and Kirouac, 2012). Cortisol has ubiquitous physiological effects throughout the body, and has been proposed to play a key role in daily cognitive and behavioral functions. Disruption of its rhythms have been implicated in the etiology of a variety of physical and mental health disorders (Adam et al., 2017). The significance of adrenal glucocorticoids to peripheral circadian rhythms have been demonstrated, as elimination of the adrenal glands in rats caused disruption of clock gene expression in the kidneys and corneas (Pezük et al., 2012). Interestingly, the adrenalectomy did not have a significant impact on the SCN, pituitary gland, or lungs of the rats, but introduction of hydrocortisone following adrenalectomy did have a significant impact on all circadian gene expression in each of these tissues (Pezük et al., 2012).

The blood concentration of cortisol, the human glucocorticoid stress hormone, varies across the 24 h day and possesses significant diurnal rhythms. It reaches its peak during the early morning, decreases during the day, and starts to rise at late night (Hoshino et al., 1987; Van Cauter et al., 1996). The phase and levels of cortisol can be used as reliable indicators for the endogenous circadian clock (Désir et al., 1980; James et al., 2007). The circadian clock could also be responsible for regulating the cortisol awakening response, an increase of cortisol within the first hour after awakening that is separate from the cortisol increase during the second half of the night (Vargas and Lopez-Duran, 2020). Circadian misalignment can cause deregulated cortisol production in neurotypical individuals. Even one night of sleep loss can elevate cortisol concentration, notably during the early morning and evening hours (Wright et al., 2015). Interestingly, there is some evidence for the absence of feedback on the SCN from the HPA axis (Oster et al., 2006). An abnormal central pacemaker stimulating the HPA axis at abnormal intervals with no feedback may cause abnormal cortisol profiles.

A number of studies suggest abnormalities in circadian rhythms of cortisol in ASD individuals, but the severity and type vary greatly (Yamazaki et al., 1975; Hill et al., 1977; Corbett et al., 2006). Aberrant rhythmic patterns of cortisol were found to be associated with lower functioning autistic children (Jensen et al., 1985; Hoshino et al., 1987). Additionally, one study found significantly decreased cortisol in ASD children, but elevated ACTH levels compared to typically developing subjects (Curin et al., 2003). Subsequently, the same researchers found a delayed cortisol response to artificial ACTH stimulation in ASD children compared to controls (Marinović-Curin et al., 2008). Another investigation found elevated ACTH and β-Endorphin (a hormone released concurrently with ACTH) in ASD individuals, but no difference in cortisol levels (Tordjman et al., 1997). On the contrary, one study measuring total daily urinary cortisol secretion found no abnormalities between typically developing individuals and ASD individuals (Marinović-Curin et al., 2008). This suggests that while measured cortisol levels and rhythms may be abnormal in ASD individuals, total cortisol output may be similar to typically developing individuals. The variable results across studies could be caused by the differences in investigation methods, size of the sample groups, and variation in determined cognitive function (low functioning vs. high functioning). A comprehensive future examination of the cortisol circadian rhythm in ASD individuals with larger sample sizes, standardized measurement methods, controlled environments, and age/gender diversification is warranted.

There is also evidence of abnormal sensitivity to stress in ASD individuals related to increased variability of cortisol rhythms (Corbett et al., 2009). One study examined cortisol response to amicable social interaction with a confederate among younger and older ASD children compared to neurotypical children. The investigators found a significant increase in cortisol levels for older ASD children compared to younger ASD children; this significant difference was not present in neurotypical individuals (Corbett et al., 2010). This difference could be explained by an awareness of social limitations among older ASD individuals, or a learned negative threat response to what would commonly be perceived as a neutral or positive social stimulus, leading to an increased cortisol response. Regardless of the etiology for the heightened response, the findings suggest increased susceptibility of the cortisol rhythm to external social zeitgebers in ASD children. Given the evidence for ASD symptoms impacting the cortisol circadian rhythm, the question remains if this relationship is unilateral, or can an aberrant circadian system in ASD individuals exert an atypical influence on cortisol levels and exacerbate negative symptoms. There is some intriguing evidence that abnormalities of cortisol circadian rhythms may be a function of ASD symptoms. A study in 2006 investigated salivary cortisol response to a disturbing non-social stimuli (mock MRI) in ASD individuals (IQ mean = 77) and neurotypical individuals, and found a strikingly significant cortisol increase in relation to the controls that exhibited a mean decrease in cortisol levels (Corbett et al., 2006). The finding of increased HPA response to a non-social stressor (blood draw) in ASD individuals compared to matched controls was replicated across variable measurements of cortisol including salivary, urinary, and serum (Spratt et al., 2012). A follow up to Corbett et al. (2006) examined cortisol response to a stressor as well as a subsequent exposure to the same stressor, and found increased circadian variability of cortisol in ASD individuals as well as increased cortisol levels in the evening following stressor exposure. This suggests the aberrant cortisol rhythm of ASD individuals may be more susceptible to entrainment by external non-social zeitgebers (Corbett et al., 2009). While there is evidence of abnormal circadian cortisol profiles in ASD individuals, the extent of the relationship and underlying mechanisms remains unclear and warrants further study.

#### Serotonin

Serotonin is produced in the central nervous system and duodenum. As serotonin cannot cross the blood-brain barrier, central and peripheral serotonergic systems are thought to be anatomically ad functionally separated (Hery et al., 1977; Ebert-Zavos et al., 2013). Serum serotonin levels exhibit diurnal variations, with a peak early in the morning and a trough in the midafternoon and during sleep (Wirz-Justice et al., 1977; Kwon et al., 2018). The diurnal oscillations of serotonin are affected by meal intake or fasting and are blunted in obese individuals (Ebert-Zavos et al., 2013; Kwon et al., 2018). An earlier study detected another peak of serotonin in the early evening (Sarai and Kayano, 1968).

In the brain, serotonin (5-hydroxytryptamine/5-HT) is synthesized in a stepwise manner from the amino acid tryptophan with two enzymes, tryptophan hydroxylase and aromatic amino acid decarboxylase (AAAD) respectively, through the intermediate, 5-hydroxytryptophan. Following release into the synaptic cleft, serotonin is retaken back into the neuron by its transporter, 5-hydroxytryptamine transporter (5HTT/SERT), or signals via one of 15 known receptors. Serotonin can also signal through monoaminylation that has been described in the monoamines 5-HT, histamine, dopamine, and norepinephrine (Walther et al., 2003; Farrelly et al., 2019). 5-HT neurons innervate into and have regulatory control on both the SCN and the intergeniculate leaflets (IGL) (Meyer-Bernstein and Morin, 1996; Glass et al., 2000, 2003). Serotonin is integral to the regulation and development of neural systems of the brain; including neural cell growth, differentiation and development of synaptic processes (D’Amato et al., 1987; Cases et al., 1995). An imbalance of serotonin negatively affects the neocortical excitation/inhibition balance, sensory stimulus perception and social communication. Abnormal serotonin levels seem to affect the synaptic processes in the sensory cortices during the developmental period (Bennett-Clarke et al., 1994; Cases et al., 1996). Abnormalities in the 5-HT system have also been associated with disruption of the mammalian circadian system and sleep–wake cycles during development (Paulus and Mintz, 2012).

The primary resource of serotonin required for development of the forebrain in the fetus is the tryptophan concentration in the placenta of the mother (Bonnin et al., 2011). Sufficient concentrations of serotonin during the prenatal and perinatal period is a determining factor for the normal regulation of the neural system, and abnormal serotonin concentration might be integral to development of ASD. Differential levels of serotonin synthesis during the stages of development underscores the importance of serotonin in the structural development of the brain in ASD individuals (Chugani et al., 1999). Human and animal studies have found that disruption of the 5-HT system during development is particularly catastrophic to phenotypic behavioral function (Sundström et al., 1993; Anderson et al., 2002; Chen et al., 2015). Proponents of the 5-HT/brainstem theory in ASD pathogenesis have proposed manifestation of ASD as a cascade of events with multiple entry points, rather than a singular devastating event. A stepwise mechanism of this cataclysmic cascade has been proposed, with particular emphasis on a mutual disturbance of the 5-HT system and the mammalian circadian system causing downstream ASD behavioral manifestation and comorbid impairments (Takumi et al., 2020).

Studies using various methodologies from biochemical analysis, genetics, neuroimaging and pharmacology have established the abnormalities of the serotonin system in ASD (Muller et al., 2016). It is well established that serotonin levels are elevated in ASD individuals (Hoshino et al., 1984; Cook et al., 1990; Gabriele et al., 2014). One of the first markers observed in autistic children was excessive levels of serotonin present in the blood plasma and more than 25% of autistic children exhibit this aberrant level of serotonin (Gabriele et al., 2014). In children diagnosed with autism, serotonin secretion is unusual, its synthesis was significantly elevated throughout late development, and the levels of serotonin correlated with the severity of autistic symptoms (Chugani et al., 1999; Abdulamir et al., 2018). However, in another study, seven ASD boys exhibited reduction in their serotonin production in left frontal cortex and thalamus, whereas one girl was unaffected. High levels of serotonin were found in the contralateral dentate nucleus of all the autistic boys (Chugani et al., 1997). High blood serotonin was found in an analysis of studies comparing ASD children to typically developing children (Gabriele et al., 2014). This further reinstates the notion of excessive serotonin in ASD children. There is some evidence of serotonin treatment returning behavior and brain function to a more typical state in an ASD mouse model (Nakai et al., 2017). Maintaining the right concentration of serotonin rescued normal conditions from autistic symptoms in mice (Nakai et al., 2017). Furthermore, a polymorphic variant identified at the site of serotonin transporter gene could result in aberrant serotonin concentration in thalamo-cortical projections (Tordjman et al., 2001).

The serotonin synthesis pathway, its molecular interactions, and the genes responsible for these interactions have been investigated in relation to the development of ASD. There is some evidence of serotonin synthesis capacity abnormalities in developing ASD individuals (Chugani et al., 1997, 1999). Decreased transporter binding of serotonin has also been demonstrated in both ASD children and adults (Makkonen et al., 2008; Nakamura et al., 2010), but there is also contradictory evidence of no significant reduction in individuals with Asperger’s Disorder (Girgis et al., 2011). Receptor binding has also been found to be reduced in ASD individuals (Murphy et al., 2006; Beversdorf et al., 2012), again with contradictory evidence among Asperger’s individuals (Girgis et al., 2011). The genes for the serotonin pathway enzymes (tryptophan hydroxylase and AAAD), its transporter (5HTT/SERT), and its receptors have all been studied as candidates for ASD pathogenesis (Makkonen et al., 2008; Nakamura et al., 2010). The gene *SLC6A4* encodes the serotonin transporter (SERT), and significant variation in allele transmission to progeny of the locus HTTLPR within *SLC6A4* has been examined in ASD; particularly an increased rate of the short allele relay compared to its long form (Devlin et al., 2005). An interesting amino acid substitution in *SLC6A4*, Gly56Ala, has been associated with certain behaviors in ASD, including compulsiveness and increased sensory aversion (Sutcliffe et al., 2005). In mice, the Gly56Ala mutation caused inhibition of social behaviors and impaired multisensory processing (Veenstra-VanderWeele et al., 2012; Siemann et al., 2017). Furthermore, there is evidence of an association between high-expressing SERT genotypes and tactile hypersensitivity in ASD individuals (Schauder et al., 2015). Regarding the enzymes responsible for 5-HT synthesis, the brain specific gene for tryptophan hydroxylase, *TPH2*, has been manipulated in mouse models extensively. When knocked out, *TPH2* null mice showed decreased vocalizations and interactions with social odors, deficits in social memory, impaired motor control, and cognitive inflexibility (Alenina et al., 2009; Del’Guidice et al., 2014; Mosienko et al., 2015). Concerning receptors, there is little evidence of malfunction in ASD. However, a few mouse models have found significant social deficits with manipulation of 5-HT1a, 5-HT1b, and 5-HT3a (Saudou et al., 1994; Smit-Rigter et al., 2010). Beyond the enzymes of the 5-HT pathway, its transporter, and its receptors, there is some evidence of malfunction in regulatory molecules and their interaction with serotonin; particularly monoamine oxidase A, the protein responsible for metabolizing 5-HT, and integrin B3 (Carneiro et al., 2008; Bortolato et al., 2013; Whyte et al., 2014). Despite intensive investigation, the mechanism of serotonin pathology in ASD remains unclear and warrants further investigations.

## Circadian Dysfunction and ASD Pathogenesis

It has been long recognized that deficits in temporal processing are fundamental in autism (Hermelin, 1972; Ornitz et al., 1972). Individuals with autism have trouble perceiving the passage of time (Martin et al., 2010; Brenner et al., 2015). Even high-functioning autism patients have a poor intuitive sense of time, and temporal information processing is disrupted (Boucher, 2001; Doenyas et al., 2019). The “weak coherence” hypothesis proposes there is a deficit or alternate pathway for neural information processing in ASD children. In typical brain development, coherence is present in the timing system, whereas in autism, coherence is out of phase and possibly responsible for social behavior deficits (Happé and Frith, 2006). The “temporal binding deficit” hypothesis proposes abnormal visual processing in ASD is due to the aberrant pattern of gamma waves which could partially explain abnormal neurobehavioral function (Brock et al., 2002). The “social timing hypothesis” proposes that biological oscillators are essential for neural information processing, and impairments in any of these oscillators would have physiological and psychological consequences. The timing deficits in ASD could be derived from pathological variations in the structure and function of clock-related genes (Wimpory et al., 2002). In support of this hypothesis, several lines of evidence indicate the dysfunction of circadian timing is associated with ASD. As aforementioned, abnormal diurnal profiles of cortisol, melatonin and abnormal sleep–wake cycles indicate underlying impairments of the circadian system in the ASD patients (Geoffray et al., 2016). In addition, here we discuss epidemiological studies linking the incidence of autism to birth seasons, clock gene polymorphisms in ASD and the role of the mTOR pathway as a common regulator of circadian rhythms and ASD pathogenesis.

### Epidemiological Evidence Indicating the Involvement of Birth Timing Factors in ASD

Epidemiological studies have linked the incidence of autism to birth seasons. In Canada, children born in spring and summer are more susceptible to development of autism than children born in winter (Konstantareas et al., 1986). In Israel, higher frequencies of ASD are found in babies born in March (18%) and August (20.2%) than babies born in February (7.6%) (Barak et al., 1995). In a more recent study in Israel, the highest incidence of ASD was found in children born during the month of May (10.3%) (Shalev et al., 2017). Another study in Italy found that some ASD children exhibited a higher degree of sleep problems when the season changed from winter to spring (Giannotti et al., 2006). The correlation between birth months and the development of autism may indicate a role for photoperiod in determining the ontogeny of the individual (Castrogiovanni et al., 1998). Many factors change with seasons. For example, the changing weather (temperature) in different seasons may be associated with different incidence of viral infection during pregnancy.

Among the many factors changing with seasons, a major factor is photoperiod (day length), which has a significant impact on the circadian clocks (Porcu et al., 2018). The durations of light and darkness in a 24-h cycle significantly influence the dynamics of circadian gene expression in different systems (Sumová et al., 2002). Rhythmic gene expression in the SCN are sculpted by the length of photoperiods. Significant differences in synchronization of clock cells and patterns of spatial clock gene expression are found between longer and shorter photoperiods (Sumová et al., 1995; Evans et al., 2013). The secretion of neuroendocrine hormones according to the biological day and night is aligned by the circadian oscillator to the changes in the environmental photoperiod (Wehr, 1998). In addition to fine-tuned circadian output by the SCN, the photoperiod also regulates the phase and levels of cortisol, melatonin and prolactin. Prolonged duration of nighttime is characterized by increased synthesis of cortisol, melatonin and prolactin and shorter nighttime periods are characterized by decreased synthesis (Wehr, 1998). Duration of the photoperiod also affects neural development and functions of offspring. Variation in the photoperiod moderates the function and electrical properties of the serotonin neurons present in the dorsal raphe nuclei of the mouse brain (Green et al., 2015). Functional properties of serotonin neurons are regulated by melatonin signaling. Firing rate and levels of the neurotransmitters serotonin and norepinephrine are altered by the duration of the light/dark cycle (Green et al., 2015). Environmental signaling of light dictates the synthesis of glucocorticoids, and timing of light exposure influences functioning of the HPA axis as well as subsequent levels of stress (Dijk et al., 2012).

Availability of nutrition, inadequate vitamin supply and infection rates vary between seasons, and could also be partially responsible for the seasonal discrepancies in ASD birth rates (Gillberg, 1990). Low birth weight, a risk factor of ASD, has been associated with season of birth (Losh et al., 2012). The birth weight of infants varies according to the season (Doblhammer and Vaupel, 2001; Day et al., 2015). Individuals born in summer had higher mean birth weight, later pubertal development and taller adult height compared to those born in all other seasons. Concordantly, those born in winter showed directionally opposite differences in these outcomes. One interesting hypothesis proposed to explain the variation in ASD rates is the availability of vitamin D to the mother (Grant and Soles, 2009). The photoperiod during the post-natal period mediates the metabolic profile and increases body weight of adults in rat models (Uchiwa et al., 2016). Regarding light-exposure, the percentage of prevalence of autism is higher in congenitally blind children (more than 30%) (Jure et al., 2016) than children with auditory impairments (1 in 59) (Szymanski et al., 2012). These findings demonstrate the association between light and its timing with the development of the neural communication system.

### Clock Gene Polymorphisms in ASD

As aforementioned, the molecular circadian clock is driven by TTFLs consisting of about a dozen clock genes in mammals (Figure 1). These clock genes are increasingly found to play fundamental roles in different physiological systems beyond their timing functions. In a mouse model, *Npas2* (–/–) caused impairments in complex emotional memory, but not non-emotional memory (Garcia et al., 2000). Another *Npas2* (–/–) mouse model showed NPAS2 is critical for non-REM sleep homeostasis and caused a reduction in total sleep time in male mice, an interesting comorbidity noted in ASD investigations (Franken et al., 2006). In humans, the protein variant NPAS2 471 Leu/Ser has been implicated in seasonal affective disorder (SAD) and diurnal preference (Johansson et al., 2003). In the repressing limb of the TTFL, PER, CRY, and CK1e form a complex in the cytoplasm, translocate across the nucleus to inhibit binding of CLOCK:BMAL1 or NPAS2:BMAL1, and downregulate transcription of both the *period* and *cryptochrome* genes (Ye et al., 2014). PER1 has been shown to have an instrumental role in cell growth and DNA damage control in human cancer cells (Gery et al., 2006). The PER1 protein also interacts with the checkpoint proteins, ATM and CHK2, regulating DNA repair and cellular apoptosis (Gery et al., 2006). Two rare variants in PER3 in humans with familial advanced sleep phase are associated with seasonal depressive traits (Zhang et al., 2016).

Increasing evidence supports the association between clock gene variants and ASD. Evidence for a genetic basis of timing in communication was originally provided by *Drosophila* studies. The *Drosophila Per* gene was the first identified clock gene and *Per* mutations disrupt the fly’s circadian rhythms (Konopka and Benzer, 1971). In addition to circadian disruption, *Per* mutations also affect the rate of sound production of the male fly’s courtship song, a primary way of communication that leads to mating (Konopka et al., 1996). In recent decades, human genetic studies of autism have identified single-nucleotide polymorphisms and *de novo* loss-of-function variants of multiple clock genes, indicating functional abnormality of these genes (Table 2). There is evidence that genes with direct influence on the mammalian circadian rhythm are highly variable in ASD individuals (Yang et al., 2016). A number of polymorphisms located within *Npas2* were identified in ASD individuals; however, only a cytosine/thymine SNP in intron 3 (NPAS2_X3_C_T) remained significant following statistical analysis (Nicholas et al., 2007). A few mutations in *Per1* were identified in ASD individuals; however, only a cytosine → guanine SNP (Per1_rs885747), and a cytosine/adenine SNP (*Per1*_rs6416892), remained significant following statistical analysis (Nicholas et al., 2007). A proline/alanine substitution at amino acid 1228 in PER2 and an arginine/glutamine substitution at amino acid 366 in PER3 were shown to negatively affect gene function; implicating PER2 and PER3 in the pathogenesis of ASD via gene expression control through the E-box (Yang et al., 2016). Also, in the repressing limb of the TTFL, REV-ERBα/β (NR1D1/2), encoded from *Nr1d1*/*2*, respectively, inhibits transcription of the activating genes *Bmal1* and *Nfil3*. *Nfil3* encodes NFIL3, a protein that upregulates production of RORα/β, and in turn RORα/β activates transcription of *Bmal1* and *Nfil3* (Preitner et al., 2002; Ueda et al., 2005). Aberrant function of RORα, possibly as a result of mutations in *Nr1d1*, has been implicated in abnormal ASD brain development (Goto et al., 2017). While the above findings are interesting and warrant investigation, polymorphisms in clock genes can only explain a small number of the abnormalities found concurrently between dysfunctional circadian related proteins and ASD phenotypes.

**TABLE 2:** Clock gene polymorphisms in ASD.

Clock genes	Chr	Location	SFARI gene and score	Findings	References
*NPAS2*	2	NC_000002.12 (100820139..100996829)	Yes, Score 3	Association analysis in an AGRE cohort revealed two Npas2 significant selected markers. Rs1811399 C > A (*p* = 0.018), and NPAS2-X3-C-T T > C (*p* = 0.028)	Nicholas et al., 2007
*PER1*	17	NC_000017.11 (8140470..8156360, complement)	Yes, Score 3	Association analysis in an AGRE cohort revealed two Per1 significant selected markers. Rs885747 C > G (*p* = 0.047), and rs6416892 C > A (*p* = 0.042)	Nicholas et al., 2007
*PER2*	2	NC_000002.12 (238244038..238290102, complement)	Yes, Score 2	A *de novo* loss-of-function variant in the PER2 gene was observed in an ASD proband from the Simons Simplex Collection in Iossifov et al. (2014). Yuen et al. (2017) identified additional PER2 variants by whole genome sequencing in four ASD families, including a de novo LoF variant in a simplex family from the ASD: Genomes to Outcome Study cohort.	Iossifov et al., 2014 Yuen et al., 2017
*PER3*	1	NC_000001.11 (7784285..7845181)	No	Base change c.1361G > A causing amino acid change p.R366Q considered disease causing in 1/28 ASD individuals with sleep disturbance.	Yang et al., 2016
*ClOCK*	4	Chromosome 4, NC_000004.12 (55427903..55547138, complement)	No	Base change c.2551A > G causing amino acid change p.H542R considered disease causing in 1/28 ASD individuals with sleep disturbance. SNP number = rs3762836	Yang et al., 2016
*ARNTL*	11	NC_000011.10 (13276552..13387268)	No	Base change c.38G > C causing amino acid change p.S13T considered disease causing in 1/28 ASD individuals without sleep disturbance	Yang et al., 2016
*ARNTL2*	12	NC_000012.12 (27332836..27425813)	No	Base change c.1418T > C causing amino acid change p.L473S considered disease causing in 1/28 ASD individuals without sleep disturbance	Yang et al., 2016
*NR1D1*	17	NC_000017.11 (40092793..40100589, complement)	Yes, Score 3	Base change c.58A > C, c.1031 A > C, c.1499G > A causing amino acid change p.S20R, p.N344T, p.R500H, respectively, considered disease causing in ASD individuals	Yang et al., 2016; Goto et al., 2017
*RORA*	15	NC_000015.10 (60488284..61229302, complement)	Yes, Score S	Allele frequencies of rs4774388 showed significant overrepresentation of T allele in patients compared with controls in Sayad et al. (2017). Increased DNA methylation and decreased gene expression of Rora in autistic co-twin than undiagnosed co-twin and unaffected controls in Nguyen et al.	Sayad et al., 2017 Nguyen et al., 2010
*RORB*	9	NC_000009.12 (74497335..74693177)	Yes, Score 1	A de novo missense variant in the RORB gene has been identified in an ASD proband from the Simons Simplex Collection by Iossifov et al. (2014) Rudolf et al. (2016) found that two individuals from patients with de novo mutations involving RORB also presented with autism spectrum disorder. Boudry-Labis et al. (2013) found that RORB was one of four genes within the minimal region of overlap in 9q21.13 microdeletion syndrome, a disorder characterized by autistic features	Iossifov et al., 2014 Rudolf et al., 2016 Boudry-Labis et al., 2013
*CSNK1E*	22	NC_000022.11 (38290691..38318084, complement)	Yes, Score 3	Two *de novo* missense variants that were predicted *in silico* to be damaging were identified in the CSNK1E gene in ASD probands from the Autism Sequencing Consortium in De Rubeis et al. (2014). Base change c.2551A > G causing amino acid change p.H542R considered disease causing by Mutation Taster analysis in three ASD individuals. SNP number = rs77945315 TADA-Denovo analysis using a combined dataset of previously published cohorts from the Simons Simplex Collection and the Autism Sequencing Consortium, as well as a novel cohort of 262 Japanese ASD trios, in Takata et al. (2018) identified CSNK1E as a gene significantly enriched in damaging *de novo* mutations in ASD cases	De Rubeis et al., 2014; Yang et al., 2016; Takata et al., 2018
*TIMELESS*	12	NC_000012.12 (56416363..56449426, complement)	No	Base changes c.1493T > C causing amino acid changes p.F498S considered disease causing in 1/28 ASD individuals with sleep disturbance	Yang et al., 2016

### A Role for the mTOR Pathway in Circadian Regulation and ASD Pathogenesis

The mTOR (mammalian target of rapamycin) signaling cascade integrates various intracellular signals to regulate cell growth and metabolism (Wullschleger et al., 2006). mTOR is a serine/threonine protein kinase that forms two multiprotein complexes in cells, mTORC1 and mTORC2. mTORC1 is composed of six components, mTOR, PRAS40, DEPTOR, mLST8 (Mammalian lethal with sec13 protein 8), Raptor, and the Tti1/Tel2 complex (Brown et al., 1994). mTORC2 is composed of seven components, four of which are shared with mTORC1: mTOR, DEPTOR, mLST8, and the Tti1/Tel2 complex. The other three, Rictor (rapamycin insensitive companion of mTOR), mSin1 (mammalian stress-activated map kinase-interacting protein 1), and Proctor are unique for mTORC2 (Jacinto et al., 2006; Pearce et al., 2007). The upstream regulators of mTORC1 are diverse, but can be generally grouped into four activators, which are oxygen, growth factors (e.g., insulin), amino acids (e.g., leucine and arginine), and energy (e.g., ATP), and one inhibitor, which is stress (Laplante and Sabatini, 2012). Notably, the GTPase activating protein Tuberous Sclerosis Complex (TSC) is the key negative regulator of mTORC1 by an intermediary effect on the GTPase, Rheb, which directly binds and activates mTORC1. The downstream effects of mTORC1 are also diverse but can be generally grouped into three categories, regulation of protein synthesis, regulation of lipid and nucleotide synthesis, and inhibition of autophagy. The regulatory and signaling pathways of mTORC2 are not as well defined, but generally it is regulated by growth factors (e.g., insulin or insulin-like growth factor-1) and has the downstream effect of cell survival and proliferation (Saxton and Sabatini, 2017). mTOR signaling regulates a variety of fundamental biological processes. During brain development, it regulates cell growth and differentiation, neuronal migration and differentiation, axonogenesis, axonal navigation and regeneration, dendrite growth and spine development, myelination by oligodendrocytes and Schwann cells, and autophagy (Cao et al., 2009). In the mature brain, it regulates synaptic plasticity, learning, memory, and feeding (Lipton and Sahin, 2014). Disruption of mTOR signaling has been implicated in a number of human brain diseases (Costa-Mattioli and Monteggia, 2013).

mTOR is emerging as a conserved circadian regulator (Cao, 2018). The mTORC1/eIF4E (eukaryotic translation initiation factor 4E) pathway regulates fundamental functions of the circadian clock such as entrainment, synchrony, and timing (Cao et al., 2013, 2015; Liu et al., 2018). In mammals, mTOR regulates the SCN circadian clock in three facets. First, mTORC1 signaling is part of the photic entrainment pathway in the SCN. In the SCN, light activates S6K1 by phosphorylating Thr389. S6K1 then phosphorylates ribosomal protein S6, a component of the 40S ribosomal subunit and regulates mRNA translation (Cao et al., 2010). S6K1 also phosphorylates the clock protein BMAL1 and activates translation (Lipton et al., 2015). On another branch, light-induced mTORC1 activation increases phosphorylation of eIF4E-binding proteins (4E-BPs) in the SCN, causing disinhibition of eIF4E-dependent translational initiation (Cao et al., 2008). Phosphorylation of both S6K1 and 4E-BP1 is mTORC1-dependent, because rapamycin eliminates the phosphorylation of both these targets in the SCN and regulates photic entrainment of the clock in animals (Cao and Obrietan, 2010; Cao et al., 2010). Second, mTORC1 regulates network properties of coupled circadian oscillators in the SCN by translational control of *Vip* (Vasoactive intestinal peptide). By phosphorylating and inhibiting the eIF4E repressor protein 4E-BP1, mTORC1 upregulates mRNA translation of *Vip* (Cao et al., 2013). VIP is synthesized by core SCN neurons, and following their photic input and entrainment, entrain and reset the shell SCN neurons that typically express arginine vasopressin (AVP). VIP signaling promotes synchrony of SCN cells, and increases the robustness of clock gene oscillations and clock functionality (Harmar et al., 2002; Aton and Herzog, 2005; Maywood et al., 2006). Conditional mTOR deletion in VIP neurons disrupts SCN cell synchrony and impairs circadian rhythms in mice, in a way largely similar to *Vip* mutation (Liu et al., 2018). Third, mTOR regulates autonomous clock properties in a variety of cellular circadian oscillators. Effects of pharmacological and genetic mTOR manipulation on autonomous circadian clock properties have been examined in various cellular and tissue oscillators including the SCN, fibroblasts, hepatocytes, and adipocytes. mTOR inhibition reduces amplitudes of oscillation and increases circadian period of the clock gene *Per2* expression, whereas mTOR activation shortens circadian period and augments amplitudes (Ramanathan et al., 2018), indicating the mTOR pathway regulates both central and peripheral clock properties.

Abnormal mTOR activities have been associated with several genetic forms of ASDs, including Tuberous Sclerosis Complex (TSC), Phosphatase and tensin homolog (PTEN), Hamartoma Tumor syndrome, Fragile X syndrome, RASopathies, Angelman Syndrome, Rett Syndrome, and Phelan-McDermid syndrome (Bhattacharya et al., 2012; Costa-Mattioli and Monteggia, 2013; Jülich and Sahin, 2014; Winden et al., 2018). Mutations in negative regulators of mTORC1, such as *TSC1*, *TSC2*, and *PTEN* are found in monogenic ASD (Buxbaum et al., 2007; O’Roak et al., 2012; Lipton and Sahin, 2014). In laboratory studies, mTOR dysregulation has been found in ASD derived neural progenitor cells (Alsaqati et al., 2020). Deletion of the *Tsc1* gene in Purkinje cells leads to mTORC1 hyperactivation and autism-like behaviors in mice (Tsai et al., 2012). Mice lacking the repressor of eIF4E, 4E-BP2, demonstrate increased translation of neuroligins, which are causally linked to ASD (Gkogkas et al., 2013). The increased levels of eIF4E also increase the ratio of excitatory: inhibitory synaptic inputs, social interaction deficits, and repetitive/stereotyped behaviors (Santini et al., 2013). A model has been proposed describing the relationship between synaptic proteins and translational control in ASD. The model includes proteins and protein complexes implicated in circadian control discussed in this paper such as Neurexins, Neuroligins, Shank, mTOR/4E-BP, and eIF4E (Santini and Klann, 2014). Thus, the circadian clock and autism are both regulated by mTOR signaling pathways. Dysregulation of the mTORC1/eIF4E axis disrupts the circadian clock and engenders ASD-like phenotypes in animal models, indicating potential crosstalk between the circadian clock and ASD via the mTORC1/eIF4E axis.

## Conclusion

In an era of rapidly increased prevalence of ASD, there is an urgent need to understand the mechanisms underlying ASD pathogenesis and develop new therapeutic strategies. Various physiological parameters such as circadian biomarkers, sleep/wake rhythms, neurotransmitters, language and communication, information processing and brain rhythms are associated with circadian clock function and are altered in ASD patients. Mounting evidence exists demonstrating malfunctions of the endogenous circadian timing system in ASD. Correlations exist between clock gene polymorphisms, seasonal discrepancies, and ASD. Understanding the functional importance of the circadian clock in neurodevelopment and its dysregulation in neurodevelopmental disorders may provide a novel approach to tackle ASD. Clinical treatments for ASD children can comprise an integrated approach considering physical, mental and social strategies based on highly dynamic daily rhythms in neurophysiology and behavior. The associations between circadian dysfunction and ASD can be bidirectional. Circadian clock malfunctions may be one of the many pathophysiological aspects underlying ASD pathogenesis, whereas experimental evidence demonstrating that circadian disruption can lead to neurodevelopmental disorders is still lacking. We propose it is necessary to comprehensively investigate the altered circadian patterns of the sleep/wake cycle, cortisol, melatonin and clock gene polymorphisms in ASD. The findings would not only reveal intrinsic connections between aberrant circadian timing and ASD development, but also be instrumental for applying chronotherapy-based strategies to treat the diseases.

## Author Contributions

EL and RK created the figures and tables. EL, RK, and RC wrote the manuscript. All the authors contributed to the article and approved the submitted version.

## Conflict of Interest

The authors declare that the research was conducted in the absence of any commercial or financial relationships that could be construed as a potential conflict of interest.

## Footnotes

1 www.cdc.gov

2 https://gene.sfari.org/
